# Editorial: Ecological, efficient and low-carbon cereal-legume intercropping systems

**DOI:** 10.3389/fpls.2023.1273675

**Published:** 2023-08-24

**Authors:** Ping Chen, Lingyang Feng, Feng Yang, Muhammad Ali Raza

**Affiliations:** ^1^ School of Life Science and Engineering, Southwest University of Science and Technology, Mianyang, Sichuan, China; ^2^ Institute of Advanced Agricultural Sciences, Peking University, Weifang, Shandong, China; ^3^ College of Agronomy, Sichuan Agricultural University/Sichuan Engineering Research Center for Crop Strip Intercropping System/Key Laboratory of Crop Ecophysiology and Farming System in Southwest, Ministry of Agriculture and Rural Affair, Chengdu, Sichuan, China; ^4^ National Research Center of Intercropping, Islamia University of Bahawalpur, Bahawalpur, Pakistan; ^5^ Gansu Academy of Agricultural Sciences, Lanzhou, Gansu, China

**Keywords:** intercropping, yield advantage, nutrient, nitrogen use efficiency, greenhouse gas emissions, legume, maize

The global population will reach 9.4–10.1 billion by 2050 (United Nations, 2019). Over the last number of decades, traditional agricultural production has met food demands by increasing resource input. However, excessive chemical fertilizer input results in severe environmental costs, e.g., soil acidification (Guo et al., 2010), global warming (Penuelas and Filella, 2001), water pollution (Yu et al., 2019), and finally cropland degradation, decreasing agricultural products and threatening human health (Han et al., 2016; Zhao et al., 2017). Moreover, increasing global food production by expanding cropland is unsustainable for the global ecosystem (Potapov et al., 2022). Expanding cropland also leads to the use of more chemical fertilizers and a high risk of global warming. Global warming increases yield losses to insect pests (Deutsch. et al., 2018), meaning more insecticide demands to guarantee crop production and a high risk of water pollution (Stehle and Schulz, 2015). Therefore, achieving global food security with environmentally friendly and sustainable development approaches is a great challenge in this century.

Intercropping is defined as simultaneously cultivating two or more crops on the same land (Willey, 1979). Intercropping is used worldwide to increase land productivity, to efficiently use resources (Li et al., 2020b; Li et al., 2021), to better control diseases and pests (Zhang et al., 2019; Chi et al., 2021), to suppress weeds (Gu et al., 2021), and to decrease environmental costs (Qin et al., 2013; Chen et al., 2019). Therefore, intercropping provides potential ways to achieve food security and sustainable agricultural development. In this Research Topic, we received recent studies revealing the mechanisms of yield advantages and the efficient use of resources in intercropping.

The complementary use of resources contributes to yield advantages in maize-legume intercropping (Li et al., 2020a). Raza et al. reported that optimizing the crop planting density maximizes the yield advantages of maize-soybean strip intercropping. Maize-soybean strip intercropping with a maize plant density of eight plants per square meter obtained a higher total leaf area index and total grain yield than other methods. The water equivalent ratios of intercropping are greater than one, suggesting that maize-soybean strip intercropping provides a potential way to achieve sustainable agricultural development. The optimized intercropping spares 20–50% of water and land. Maize-soybean intercropping with a N input of 250 kg N ha^−1^ obtained yield advantages (Nasar et al.). The underlying yield advantages include increased N use efficiency, e.g., N uptake efficiency and N agronomic efficiency since the N assimilatory enzymes of intercropped maize, e.g., nitrate reductase, nitrite reductase, and glutamate synthase, are more robust than the monoculture.

However, the underlying mechanisms of yield advantages of component crops in relay intercropping are different. Chen et al. revealed the mechanism for intercropped maize over-yielding in a low radiation area. The net yield of intercropped maize can be increased by 2.1 Mg ha^−1^ *via* the use of dense cultivation and high N input with plow tillage compared with normal farming practice. The over-yielding of intercropped maize mainly derives from an improved leaf area index (LAI) and net photosynthetic rate (Pn). Similarly, Zheng et al. showed that straw incorporation increases the aboveground N uptake and nitrogen recovery efficiency of intercropped soybean by 43.7% and 76.8%, respectively, compared with straw removal. In particular, straw incorporation at 30 kg N ha^−1^ achieved the greatest aboveground N uptake and nitrogen recovery efficiency compared with other N treatments. Although straw incorporation remarkably promotes CO_2_ emission, the accumulated CO_2_ emission of straw incorporation was lowest at 30 kg N ha^−1^.

Legumes’ performance in strip and relay intercropping differs (Zhang et al., 2023). In relay intercropping, the recovery growth of legumes benefits their yield advantage (Wu et al., 2021). In maize-peanut strip intercropping, the crop planted later, e.g., peanut, suffers from the shade of maize (Chen et al., 2020). Lu et al. pointed out that optimizing crop configurations increases light use and obtains yield advantages in maize-peanut strip intercropping. Although intercropped peanut suffers from the shade of maize, which decreases the leaf functional traits, intercropped peanut in eight rows allows higher light energy utilization than intercropped peanut in four or two rows. Previous studies reported that intercropped maize with legumes increases the usage efficiency of resources by optimizing crop root distribution and strengthening nutrient acquisition (Chen et al., 2017; Zheng et al., 2021; Zheng et al., 2022). Surigaoge et al. pointed out that cereal-legume intercropping improves soil nutrient cycling. Plant litter is decomposed more quickly in maize-peanut intercropping than in maize-soybean intercropping. Although N addition promotes plant litter decomposition, maize-peanut intercropping achieved a higher decomposition rate than maize-soybean intercropping. Moreover, a trade-off in yield advantage is observed in maize-wheat relay strip intercropping under rainfed conditions(Hussain et al.). N input contributes to a more robust yield advantage by strengthening the yield advantage of intercropped wheat in the border rows. Specifically, the yield advantage of intercropped wheat in the border rows is mainly attributed to a higher number of ears in the unit area. In contrast, yield disadvantage is obtained in intercropped maize due to the lower kernel number and thousand-grain weight of maize in the border rows compared with maize alone.

The practice of intercropping is not limited to staple crops; intercropping of vegetables or forage grass is also valuable (Stoltz and Nadeau, 2014).Pereira et al. pointed out that vegetable intercropping can mitigate greenhouse gas (GHG) emissions. Collard greens-spinach and collard greens-chicory intercropping decreased GHG emissions by 31% compared with the corresponding monoculture. Tahir et al. reported that a full mixture of legume-grass increases farmland productivity. The mixture is beneficial in improving the soil enzyme activity and in increasing the soil nutrient content. In return, the improved growth of forage leads to higher levels of crude protein than the monoculture, and the crude protein content of the mixture increases with increasing N input.

This Research Topic confirms the potential of intercropping to achieve food security using environmentally friendly approaches. Advisors and farmers can refer to this knowledge to optimize their decision-making in crop management and to improve food security and quality.

## Author contributions

PC: Writing – original draft, Writing – review & editing. LF: Writing – original draft, Writing – review & editing. FY: Writing – original draft, Writing – review & editing. MR: Writing – original draft, Writing – review & editing.
